# Risk factors associated with brachial–ankle pulse wave velocity among peritoneal dialysis patients in Macao

**DOI:** 10.1186/1471-2369-13-143

**Published:** 2012-11-01

**Authors:** Ding-Wei Kuang, Chiu-Leong Li, Un-I Kuok, Kin Cheung, Weng-In Lio, Jing Xin

**Affiliations:** 1 Divion of Nephrology, Centro Hospitalar Conde de São Januário, Macao SAR, PR China; 2 Divion of Nephrology, Huashan Hospital, Fudan University, Shanghai, PR China

**Keywords:** Arterial stiffness, Pulse wave velocity, Cardiovascular disease, Peritoneal dialysis

## Abstract

**Background:**

Cardiovascular disease is the leading cause of mortality among peritoneal dialysis (PD) patients in Macao. Increased arterial stiffness determined by pulse wave velocity (PWV) has been established as an independent predictor of cardiovascular mortality in end-stage renal disease patients. The present study aims to investigate the relationship between arterial stiffness and its associated risk factors in chronic PD patients.

**Methods:**

A total of 96 chronic PD patients (48 males/48 females) were included in the cross-sectional study. Arterial stiffness was assessed by brachial-ankle PWV (baPWV). Patients were divided into two subgroups according to mean baPWV value. On enrollment, clinical characteristics and biochemical parameters were collected.

**Results:**

Compared with low baPWV group patients, high baPWV group patients were significant older (*p*<0.001) and more likely to have a high proportion of female gender (*p*=0.004) as well as previous CVD history (*p*=0.008). Serum albumin, pre-albumin levels and residual renal creatinine clearance (CCr) were significantly lower but the serum ferritin level was significantly higher in high baPWV group patients than in low baPWV group patients (all *p*<0.01). BaPWV was positively associated with age (*r*=0.534, *p*<0.001), Charlson comorbidity index (*r*=0.350, *p*<0.001) and serum ferritin level (*r*=0.340, *p*=0.001). Meanwhile, baPWV negatively correlated with serum albumin (*r*=−0.479, *p*<0.001), pre-albumin levels (*r*=−0.320, *p*=0.003) and residual renal CCr (*r*=−0.177, *p*=0.048). Age-adjusted partial correlation test found a significant correlation between baPWV and CRP (*r*=0.462, *p*<0.001). Multivariate regression analysis showed that baPWV was independently associated with age (*p*<0.001), serum albumin level (*p*=0.015), CRP (*p*=0.019) and residual renal CCr (*p*=0.045).

**Conclusion:**

Arterial stiffness, assessed by baPWV, had an independent correlation with age, serum albumin level, CRP level and residual renal CCr among PD patients in Macao.

## Background

Besides Hong Kong, Macao is the other special administrative region (SAR) belongs to the People’s Republic of China. As the only government hospital in Macao, Centro Hospitalar Conde de São Januário is responsible for all of the peritoneal dialysis (PD) patients. Benefitting from the superior end-stage renal disease (ESRD) funding system provided by Macao SAR, almost all the resident patients on chronic dialysis can get free treatment including dialysis and medication. Different from Hong Kong, there is no PD-first policy in Macao. Nevertheless, PD has been rapidly developed during the past decade in Macao with about 40% of the PD penetration rate in chronic dialysis patients [1].

Cardiovascular disease (CVD) is the leading cause of mortality and morbidity among patients with ESRD on chronic dialysis [2]. According to U.S. Renal Data System and Hong Kong Renal Registry reports, CVD accounts for approximately 40% mortality in dialysis patients and also is the main cause of hospitalization [3,4]. In keeping with these facts, data from the Macao Renal Registry show that 36.2% of the mortality in PD patients is attributable to CVD. Currently, many available studies have shown that increased arterial stiffness, which can be examined by pulse wave velocity (PWV), is a powerful and independent predictor of all-cause and cardiovascular mortality in ESRD patients [5,6]. Available study [7] has clearly shown that the eGFR slope was negatively associated with baPWV in patients with chronic kidney disease stages 3 to 5. Moreover, higher baPWV was independently associated with progression to commencement of dialysis or death.

Generally, carotid-femoral PWV (cfPWV), calculated on the basis of pulse transit time and the distance travelled by a pulse between carotid artery and femoral artery, is considered as a well-established index of central arterial stiffness [8]. However, there are some obvious limitations for routine use cfPWV measurement in clinics [9]. Firstly, it is somewhat difficult for clinical operator to use pressure transducers on target arteries. Additionally, some subjects may feel uncomfortable and generally hesitate to exposing inguinal area during the acquisition of femoral pressure waveforms. Recently, the brachial–ankle PWV (baPWV) technique has been developed due to its simple way of measurement by only wrapping the four extremities with blood pressure cuffs. Since most CVD events in PD patients are related with peripheral arteries but not aortic artery, it is important that baPWV reflects both central and peripheral arterial stiffness [10]. However, available reports on arterial stiffness in PD patients were limited and cfPWV was used for assessment in all studies [11-14]. The clinical value of baPWV in chronic PD patients has not yet been fully evaluated.

Although an increasing PWV of PD patients is reported to be significantly associated with age, malnutrition, pulse pressure, and peritoneal transport status by a single-center study in North China [11,12], it has not yet been elucidated whether there are some dissimilar impact factors on arterial stiffness among Macao PD patients who live in South China with inherent variations in different geographic characteristics, primary diseases, and underlying medical funding systems. Therefore, this present study aims to investigate the relationship between baPWV and its associated risk factors among chronic PD patients in Macao.

## Methods

### Patients

We studied all chronic PD patients (n=107) in our center in this cross-sectional study. All patients were undergoing continuous PD therapy. Exclusion criteria were:(1) treatment time <3 months prior to enrollment; (2) age younger than 18 years; (3) presence of clinically overt congestive heart failure (NYHA class III-IV); (4) peritonitis less than 1 month before the study; (5) persistent hypotension despite pharmacological therapy which was defined as systolic blood pressure (BP) <90 mmHg or diastolic BP <60 mmHg; (6) unwillingness to participate in our study. Finally, total 96 patients were included in the study. On enrollment, demographic and clinical data were collected, including age, gender, height, weight, body mass index (BMI), BP, presence of diabetes mellitus, medication history and etiology of ESRD. The causes of ESRD were as follows: chronic glomerulonephritis (n=38), diabetes mellitus (n=34), essential hypertension or ischemic nephropathy (n=21), obstructive nephropathy (n=1), lupus nephritis (n=1), polycystic kidney disease (n=1). Of total 96 patients, 32 patients were ongoing automatic PD (APD) therapy and 35 patients were using low glucose degradation product peritoneal dialysis fluid (Low-GDP PDF). Charlson comorbidity index (CCI) was scored as described by Beddhu S et al. [15]. Hyperlipidemia was defined as diagnosed according to Adult Treatment Panel III criteria or use of statins. This study was approved by the Ethical Committee of Centro Hospitalar Conde de São Januário and written informed consent was obtained from all participants.

### Biochemical analysis

Fasting blood samples were collected in the morning. Measurements were performed using routine laboratory methods for such serum parameters as creatinine, calcium, phosphate, albumin, pre-albumin, total cholesterol, triglyceride, low- and high-density lipoprotein, ferritin, hematocrit and hemoglobin. Serum C-reactive protein (CRP) was measured by a high-sensitivity commercial assay. Serum intact parathyroid hormone (iPTH) was measured by Nichols immunoradiometric assay. Blood, urine and dialysate samples were collected in order to calculate weekly Kt/V and creatinine clearance (CCr).

### PWV measurement

The baPWV was assessed using VP-1000 vascular profiler (Nippon Colin Ltd., Komaki City, Japan), which allowed on-line pulse wave recording and automatic calculation of PWV. Briefly, baPWV was calculated from the equation: (D1 - D2)/T. D1 is the distance between the heart and ankle, D2 is the distance between the heart nd brachium, and T is the transit between the right brachial arterial wave and right tibial arterial wave. The distances between the sampling points are automatically calculated from the patient’s height and are divided by the time interval for the waveform from each measuring point. The baPWV was performed in PD patients with empty abdomen after drainage of dialysate and at least 15 minutes supine rest. Two measurements were performed in each arm, and the average value was used for the analysis. BaPWV is used as arterial stiffness markers due to ease of measurement, reproducibility and validity in previous studies [16]. All the PWV measurements were performed by one experienced operator and the intra-observer coefficient of variation was about 1.58–3.36%. Patients were divided into two groups according to mean baPWV value: those above the mean baPWV value were in the high baPWV group, while those below the mean baPWV value were in the low baPWV group.

### Statistical analysis

Continuous variables with normal distribution were expressed as means ± standard deviation, while those without normal distribution were shown as median and interquartile range. Comparisons between the two groups were done by student’s *t* test or *χ*^2^ test. Non-parametric data were compared with using Mann–Whitney *U* test. Univariate analysis was done to explore relationships between baPWV and other variables by Pearson correlation test for normally distributed data and Spearman Rank correlation test for non-parametric data (Model 1). Age-adjusted partial correlation test (Model 2) also was performed. Step-wise multiple linear regression analysis was used to assess the independent determinants of increased baPWV. We included all significant variables with respect to the univariate analysis. Variables recognized to present clinical relevance in the current literature but not presenting statistical significance in our study were also included. A two tailed *p* < 0.05 was considered as statistically significant. All statistical analyses were performed using the SPSS statistical software 17.0 for Windows (SPSS, Chicago Davis, IL. USA).

## Results

### Patient characteristics and comparisons between two subgroups

Table 1 shows the demographic and clinical characteristics of enrolled PD patients and the comparisons between two subgroup patients divided according to the mean baPWV value. Compared with low baPWV group patients, high baPWV group patients were significantly older (*p*<0.001) and more likely to have a high proportion of female gender (*p*=0.004) as well as previous CVD history (*p*=0.008). Table 2 shows the laboratory parameters of enrolled PD patients and the comparisons between two subgroup patients. Serum albumin level (*p*<0.001), pre-albumin level (*p*=0.004) and residual renal CCr (*p*=0.008) were significantly lower but the serum ferritin level (*p*=0.009) was significantly higher in high baPWV group patients compared with low baPWV group patients. However, there were no significant differences in CCI, PD duration, BP, BMI, weekly erythropoietin dosage, rennin-angiotensin system inhibitor or β-blocker use, APD use, low-GDP PDF use, diabetic status, CRP, serum phosphate and iPTH levels between two subgroups (all *p*>0.05).

**Table 1:** Demographic and clinical characteristics of study population and comparisons between subgroups

**Variables**	**Total**	**Low baPWV Group**	**High baPWV Group**	***p *Value**
	**(n=96)**	**(n=56)**	**(n=40)**	
Female Gender	50.0%	37.5%	67.5%	0.004
Age (years)	63.92 ± 14.24	58.34 ± 14.13	71.73 ± 10.26	<0.001
CCI (score)	5.0 (2.0, 9.0)	4.0 (2.0, 8.0)	5.0 (3.0, 9.0)	0.054
PD duration (Months)	44.47 ± 27.89	40.86 ± 26.12	49.53 ± 29.80	0.134
SBP (mmHg)	135.75 ± 20.13	137.07 ± 20.58	133.90 ± 19.59	0.450
DBP (mmHg)	76.21 ± 14.58	78.39 ± 13.32	73.15 ± 15.84	0.082
MAP (mmHg)	96.06 ± 14.81	97.95 ± 13.95	93.40 ± 15.73	0.138
PP (mmHg)	59.54 ± 16.07	58.68 ± 17.08	60.75 ± 14.68	0.536
BMI (kg/m^2^)	23.44 ± 3.65	23.26 ± 3.47	23.70 ± 3.92	0.559
Dose of EPO (U/kg.week)	136.95 ± 98.18	137.35 ± 90.53	136.39 ± 109.18	0.963
RAS inhibitor use	62.5%	67.9%	55.0%	0.200
β-Blocker use	13.5%	14.3%	12.5%	0.801
Hyperlipidemia	40.6%	39.3%	42.5%	0.752
Previous CVD history	26.0%	16.1%	40.0%	0.008
APD use	33.3%	41.1%	22.5%	0.057
Low-GDP PDF use	36.5%	33.9%	40.0%	0.542
Diabetes mellitus	34.4%	32.1%	37.5%	0.586
baPWV (m/s)	21.20 ± 5.63	17.49 ± 2.41	26.39 ± 4.66	<0.001

Data were divided into two groups according to mean baPWV value.

Abbreviations: *baPWV*, brachial-ankle pulse wave velocity; *CCI*, Charlson comorbidity index; *PD*, peritoneal dislysis; *SBP*, systolic blood pressure; *DBP*, diastolic blood pressure; *MAP*, mean arterial pressure; *PP*, pulse pressure; *BMI*, body mass index; *EPO*, erythropoietin; *RAS*, renin-angiotensin system; *APD*, automatic peritoneal dialysis; *GDP*, glucose degradation product; *PDF*, peritoneal dialysis fluid.

**Table 2:** Laboratory parameters of study population and comparisons between subgroups

**Variables**	**Total**	**Low baPWV Group**	**High baPWV Group**	***p *Value**
	**(n=96)**	**(n=56)**	**(n=40)**	
Serum Albumin(g/L)	38.72 ± 4.85	40.41 ± 3.50	36.35 ± 5.48	<0.001
Pre-Albumin(g/L)	40.16 ± 10.84	42.75 ± 9.61	35.92 ± 11.53	0.004
Cholesterol(mmol/L)	4.82 ± 1.17	4.87 ± 1.35	4.77 ± 0.88	0.682
Triglycerides(mmol/L)	1.92 (0.43, 8.70)	1.79 (0.43, 5.38)	2.08 (0.89, 8.70)	0.314
LDL-cholesterol(mmol/L)	2.72 ± 1.13	2.73 ± 1.23	2.70 ± 0.97	0.909
HDL-cholesterol(mmol/L)	1.22 ± 0.44	1.25 ± 0.50	1.18 ± 0.35	0.451
Hemoglobin(g/dL)	11.17 ± 1.39	11.32 ± 1.41	10.96 ± 1.35	0.214
Hematocrit(%)	32.76 ± 4.26	33.19 ± 4.47	32.15 ± 3.91	0.239
Ferritin(ug/L)	658.89 ± 457.06	549.51 ± 343.48	812.04 ± 548.87	0.009
C-reactive protein(mg/L)	0.41 (0.10, 15.70)	0.37 (0.10, 4.46)	0.59 (0.10, 15.70)	0.104
Calcium(mmol/L)	2.40 ± 0.25	2.37 ± 0.21	2.44 ± 0.30	0.186
Phosphate(mmol/L)	1.53 ± 0.43	1.60 ± 0.44	1.44 ± 0.40	0.071
Calcium-phosphate product[(mmol/L)^2^]	3.67 ± 1.12	3.79 ± 1.18	3.49 ± 1.02	0.198
intact-PTH(pg/ml)	342.50 (11.50, 1761.00)	360.75 (11.50, 1486.00)	306.20 (56.45, 1761.00)	0.970
Kt/V (total)	2.11 ± 0.58	2.13 ± 0.68	2.09 ± 0.40	0.783
Kt/V (renal)	0.35 ± 0.48	0.43 ± 0.52	0.24 ± 0.41	0.052
CCr (total)	59.04 (42.23, 138.22)	60.58 (42.23, 138.22)	58.37 (42.51, 117.01)	0.476
CCr (renal)	6.16 (0.00, 90.00)	14.52 (0.00, 90.00)	0.46 (0.00, 75.49)	0.008

Data were divided into two groups according to mean baPWV value.

Abbreviations: *baPWV*, brachial-ankle pulse wave velocity; *LDL*, low-density lipoprotein; *HDL*, high-density lipoprotein; *PTH*, parathyroid hormone; *CCr*, creatinine clearance.

### Correlations between baPWV and related parameters

Table 3 shows the correlations between baPWV and related parameters by means of univariate analysis. In brief, Model 1 showed that baPWV was positively associated with patients’ age (*r*=0.534, *p*<0.001), CCI (*r*=0.350, *p*<0.001) and serum ferritin level (*r*=0.340, *p*=0.001). Meanwhile, baPWV negatively correlated with serum albumin (*r*=−0.479, *p*<0.001), pre-albumin levels (*r*=−0.320, *p*=0.003) and residual renal CCr (*r*=−0.177, *p*=0.048). However, no significant correlation was found between baPWV and CRP (*r*=0.182, *p*=0.076). Results of partial correlation test from Model 2 by controlling for age showed that the correlation between CCI and baPWV was missing, but a significant correlation was found between baPWV and CRP (*r*=0.462, *p*<0.001).

**Table 3:** Correlation between pulse wave velocity and related parameters

**Variables**	**Model 1**		**Model 2**	
	**Coefficient**	***P *Value**	**Coefficient**	***P *Value**
Age (years)	0.534	<0.001	-	-
CCI (score)	0.350	<0.001	0.059	0.592
PD duration (Months)	0.063	0.539	0.128	0.239
SBP (mmHg)	−0.003	0.976	0.089	0.418
DBP (mmHg)	−0.125	0.226	0.045	0.682
MAP (mmHg)	−0.083	0.420	0.070	0.522
PP (mmHg)	0.109	0.290	0.073	0.504
BMI (kg/m^2^)	0.104	0.314	0.009	0.937
Serum Albumin(g/L)	−0.479	<0.001	−0.451	<0.001
Pre-Albumin(g/L)	−0.320	0.003	−0.161	0.139
Cholesterol(mmol/L)	−0.064	0.538	−0.090	0.408
Triglycerides(mmol/L)	0.022	0.829	−0.018	0.870
LDL-cholesterol(mmol/L)	0.047	0.647	0.064	0.560
HDL-cholesterol(mmol/L)	0.003	0.974	−0.041	0.711
Hemoglobin(g/dL)	−0.106	0.306	−0.166	0.126
Hematocrit(%)	−0.078	0.451	−0.129	0.235
Ferritin(ug/L)	0.340	0.001	0.293	0.006
C-reactive protein(mg/L)	0.182	0.076	0.462	<0.001
Calcium(mmol/L)	0.071	0.494	−0.002	0.984
Phosphate(mmol/L)	−0.147	0.154	0.010	0.924
Calcium-phosphate product[(mmol/L)^2^]	−0.121	0.241	0.004	0.974
intact-PTH(pg/ml)	−0.135	0.189	−0.012	0.911
Kt/V (total)	−0.025	0.807	−0.093	0.394
Kt/V (renal)	0.110	0.288	−0.294	0.005
CCr (total)	−0.013	0.901	−0.276	0.010
CCr (renal)	−0.177	0.048	−0.301	0.005

Abbreviations: *CCI*, Charlson comorbidity index; *PD*, peritoneal dialysis; *SBP*, systolic blood pressure; *DBP*, diastolic blood pressure; *MAP*, mean arterial pressure; *PP*, pulse pressure; *BMI*, body mass index; *LDL*, low-density lipoprotein; *HDL*, high-density lipoprotein; *PTH*, parathyroid hormone; *CCr*, creatinine clearance.

### Multivariate regression analysis of determinants of baPWV

In a stepwise multiple linear regression analysis, we employed baPWV value as a dependent variable, while using age, CCI, MAP, calcium-phosphate product, serum albumin level, ferritin level, total cholesterol level, CRP and residual renal CCr as independent variables. Table 4 shows the multivariate linear regression analysis results. Age, serum albumin level, CRP and residual renal CCr were independently associated with baPWV (*p*<0.001, =0.015, =0.019 and =0.045, respectively) and together explained 51.1% of the total variance.

**Table 4:** Multiple linear regression analysis of factors associated with pulse wave velocity

	**B**	**SE**	**Standardized coefficient**	***t *value**	***P *value**
Constant	1538.293	604.077		2.547	0.013
Age	14.814	3.684	0.375	4.021	<0.001
Serum albumin	−27.891	11.233	−0.240	−2.483	0.015
CRP	53.088	22.299	0.210	2.381	0.019
CCr (renal)	−3.806	1.874	−0.169	−2.031	0.045

Abbreviations: *CRP*, C reactive protein; *CCr*, creatinine clearance.

## Discussion

Chronic PD patients are at a high risk of developing accelerated atherosclerosis, vascular stiffness and CVD incidence secondary to a multitude of traditional and uremia-specific risk factors [17]. The present study investigated the arterial stiffness and its associated factors among stable PD patients in Macao. We found that baPWV was independently correlated with patient’s age, serum albumin level and residual renal CCr.

Arterial stiffness has taken on great importance in the pathophysiology of CVD. In previous studies of general population as well as ESRD patients, increased arterial stiffness assessed by PWV was well established as an independent predictor of all-cause and cardiovascular mortality [5,18]. More recently, study by Sipahioglu et al. [19] reported that arterial stiffness was an independent risk predictor of mortality and adverse CVD outcome in PD patients. Although epidemiological data show that mortality rate in PD patients continues to decline, long-term survival remains poor. CVD accounts for most deaths, therefore, strategies aimed at reducing modifiable risk factors of CVD have been highlighted for enhancing long-term survival in PD patients [20]. Given the multivariate regression analysis findings in this study, patients’ age, as one of the independent associated factors with baPWV, cannot be modified. However, attention needs to be placed on other modifiable risk factors which are strongly correlated with increasing arterial stiffness in PD patients, including improvement of malnutrition status and prevention of residual renal function.

Hypoalbuminemia is an independent predictor of increased CVD and mortality in dialysis patients, although the exact mechanisms remain unclear. In consistent with the previous study reported by Gu et al. [12], we showed that serum albumin level was independently associated with baPWV in PD patients. Several factors may contribute to malnutrition in PD patients such as low protein or energy intakes, psychosocial factors, catabolic effects of acidosis, dialytic losses of protein or amino acids, bio-incompatibility of PDF, and infection [21]. Low serum albumin level may induce micro or clinical systemic inflammation which may play an additive role on atherosclerotic vascular disease progression [22]. Moreover, growing evidences have suggested that hypoalbuminemia was associated with increased oxidative stress which could accelerate atherosclerosis process in dialysis patients [23]. In addition, a number of studies showed that nutritional deficiencies may also play an important role on endothelial dysfunction in ESRD as well as dialysis patients [24].

Generally, various factors associated with PD procedure, such as peritonitis, exit site infection, use of bio-incompatible PDF may promote inflammation. Protein-energy malnutrition with micro-inflammation presenting in a large proportion of chronic PD patients is widely accepted to be a strong risk factor for cardiovascular mortality in this patient group [25]. Meanwhile, inflammation has been proposed to be a critical promoter of atherosclerosis, interacting with many pathophysiologic pathways to lead to vascular stiffness. Although the precise link between inflammation and CVD mortality in PD patients remains unknown, endothelial dysfunction has been proposed to play an important role in inflammation-mediated artherosclerosis [26]. CRP, which is one of the prototypic markers of inflammation, has been showed to be an important predictor of mortality and CVD death in PD patients [27]. Inconsistent with previous single-centre studies in North China [11,12], we found that CRP was an independent risk factor for baPWV. Besides the different measurement for arterial stiffness (baPWV vs cfPWV), it may also partly be explained by the higher proportion of DM (35.4% vs 30.0%) and the longer average duration of PD (44.5 months vs 9.5 months) in our studied population.

It has been clearly established that residual renal function is related to all-cause mortality and risk of cardiovascular death in PD patients [28]. The reanalysis of the CANUSA study [29] demonstrated that patient survival was linked with the magnitude of residual renal function. Each 5-L per week per 1.73 m^2^ increase in residual glomerular filtration rate corresponded to a 12% decrease in the relative risk of death. Neither peritoneal CCr nor net peritoneal ultrafiltration was associated with patient survival. In the present study, residual renal CCr, instead of peritoneal CCr, was independently associated with baPWV in PD patients. In addition to better solute clearance and volume removal, residual renal function was also associated with decreased levels of circulating inflammatory markers and free radicals, reduced BP, increased phosphorus removal, and reduced left ventricular hypertrophy [30]. Taken together, these multiple factors may contribute to improvement on vascular endothelial dysfunction and atherosclerosis.

Several limitations of this study must be taken into consideration when interpreting the data. Firstly, its cross-sectional design of the study did not allow us to determine causality. Secondly, although many potential confounding factors have been assessed, the existence of other unrecognized variables should be noted. Finally, due to the technical limitation of baPWV measurement, patients with atrial fibrillation or amputated extremity were excluded. However, these PD patients generally are relevant to high risk of arterial stiffness.

## Conclusions

In conclusion, the presented data clearly showed that arterial stiffness, which was assessed by baPWV, had an independent correlation with age, serum albumin level, CRP level and residual renal CCr in PD patients. Further scaled and prospective studies are warranted to investigate whether amelioration of malnutrition and micro-inflammatory status and preserving residual renal function might prevent the progression of arterial stiffness and reduce the CVD incidence among chronic PD patients.

## Competing interests

The authors declare that they have no competing interests.

## Authors’ contributions

KDW collected data, analyzed, interpreted data and drafted the manuscript. LCL collected data and drafted the manuscript. CK, LWI and XJ collected data, analyzed and revised the manuscript. KUI conceived the study, participated in its design and coordination and helped to draft the manuscript and had full access to all the data and assume responsibility for the integrity of the data and the accuracy of the analysis. All authors read and approved the final manuscript.

## Pre-publication history

The pre-publication history for this paper can be accessed here:

http://www.biomedcentral.com/1471-2369/13/143/prepub
